# Item response theory and factor analysis as a mean to characterize occurrence of response shift in a longitudinal quality of life study in breast cancer patients

**DOI:** 10.1186/1477-7525-12-32

**Published:** 2014-03-08

**Authors:** Amélie Anota, Caroline Bascoul-Mollevi, Thierry Conroy, Francis Guillemin, Michel Velten, Damien Jolly, Mariette Mercier, Sylvain Causeret, Jean Cuisenier, Olivier Graesslin, Zeinab Hamidou, Franck Bonnetain

**Affiliations:** 1 Quality of Life in Oncology Platform, Besançon, France; 2 Methodological and Quality of Life Unit in Oncology, University Hospital of Besançon, Besançon, France; 3 EA 3181, University of Franche-Comte, Besançon, France; 4 Department of Biostatistics, Institut Régional du Cancer Montpellier, Montpellier, France; 5 Medical Oncology Department, Centre Alexis Vautrin, Nancy, France; 6 Clinical Epidemiology and Evaluation Department, Inserm, CIC-EC, and CHU, Nancy, France; 7 Department of Epidemiology and Public Health, Faculty of Medicine, EA 3430, University of Strasbourg, Strasbourg, France; 8 Pôle Recherche – Innovations, University Hospital of Reims, Reims, France; 9 Surgery Department, Centre Georges François Leclerc, Dijon, France; 10 Gynecological and Obstetric Department, Institut Mère Enfant, University Hospital of Reims, Reims, France; 11 Public health laboratory, EA 3279, Aix-Marseille University, Marseille, France

**Keywords:** Health-related quality of life, Longitudinal analysis, Response shift, Factor analysis, Item Response Theory

## Abstract

**Background:**

The occurrence of response shift (RS) in longitudinal health-related quality of life (HRQoL) studies, reflecting patient adaptation to disease, has already been demonstrated. Several methods have been developed to detect the three different types of response shift (RS), i.e. recalibration RS, 2) reprioritization RS, and 3) reconceptualization RS. We investigated two complementary methods that characterize the occurrence of RS: factor analysis, comprising Principal Component Analysis (PCA) and Multiple Correspondence Analysis (MCA), and a method of Item Response Theory (IRT).

**Methods:**

Breast cancer patients (n = 381) completed the EORTC QLQ-C30 and EORTC QLQ-BR23 questionnaires at baseline, immediately following surgery, and three and six months after surgery, according to the “then-test/post-test” design. Recalibration was explored using MCA and a model of IRT, called the Linear Logistic Model with Relaxed Assumptions (LLRA) using the then-test method. Principal Component Analysis (PCA) was used to explore reconceptualization and reprioritization.

**Results:**

MCA highlighted the main profiles of recalibration: patients with high HRQoL level report a slightly worse HRQoL level retrospectively and vice versa. The LLRA model indicated a downward or upward recalibration for each dimension. At six months, the recalibration effect was statistically significant for 11/22 dimensions of the QLQ-C30 and BR23 according to the LLRA model (p ≤ 0.001). Regarding the QLQ-C30, PCA indicated a reprioritization of symptom scales and reconceptualization via an increased correlation between functional scales.

**Conclusions:**

Our findings demonstrate the usefulness of these analyses in characterizing the occurrence of RS. MCA and IRT model had convergent results with then-test method to characterize recalibration component of RS. PCA is an indirect method in investigating the reprioritization and reconceptualization components of RS.

## Background

Health-related quality of life (HRQoL) is a subjective clinical endpoint that has been increasingly important in health outcomes research and particularly in cancer clinical trials over the past two decades [1] as well as in breast cancer [2]. Although overall survival is still considered as the primary objective and the primary endpoint in many studies, most clinical trials now integrate HRQoL as an endpoint in order to investigate the clinical benefit for the patient.

One major objective of measuring HRQoL over time is determining the extent to which treatment toxicities or disease progression can affect patients’ HRQoL level. However, self-assessment of HRQoL is subjective, *i.e.* it is dependent on the patient's internal standards and definition of HRQoL [3-5]. As patients can adapt to disease and treatment toxicities, their health and HRQoL expectations can also change over time. These changes could result in a response shift (RS) effect [6-8].

RS can be defined as “a change in the meaning of one’s self-evaluation of a target construct as a result of: (a) a change in the respondent’s internal standards of measurement (i.e. scale recalibration); (b) a change in the respondent’s values (i.e. the importance of component domains constituting the target construct, [reprioritization]) or (c) a redefinition of the target construct (i.e. reconceptualization)” [9].

Different methods have been proposed to assess RS [10-13]. The most widely used is the “Then-test” method, which assesses patients’ pre-test HRQoL levels retrospectively. The test involves asking patients post-treatment to provide their current levels (post-test) but also their pre-test levels in retrospect (then-test). This method is based on the assumption that patients rate their HRQoL post-test and pre-test levels with the same criteria, since the assessments occur at the same time point. The recalibration component of RS should thus be taken into account when comparing post-test and then-test scores. Comparing the mean of the pre-test and then-test scores explores recalibration component of RS [12].

Statistical methods have also been investigated to detect RS. First, factor analyses have been explored to detect RS [14,15]. An alternative to investigate RS with factor analysis is the use of structural equation modeling (SEM) [11,16,17]. These models can evaluate all types of RS if they are experienced by a substantial part of individual in the population analyzed [16]. These models are based on means and covariance structures and rely on observed scores. At this time and to our knowledge, these models have never been applied to an European Organization for Research and Treatment of Cancer (EORTC) HRQoL questionnaire in order to highlight RS effect. Principal Component Analysis (PCA) is a special case of SEM. Item Response Theory (IRT) could also be considered to explore RS effect but up to now these models remain less applied and mainly through differential item functioning [18,19]. Contrary to SEM, IRT models are not based on the observed scores but directly on items answers. In fact, in SEM, the raw score is assumed to be a good representation of the latent trait (i.e. HRQoL), while in IRT the items responses play a key role and the relation between the items responses and the latent trait are not linear in IRT.

While occurrence of RS in HRQoL studies has been demonstrated [13], approaches that could reinforce the proof that each component of RS occurs should be investigate to complement the results from other methods. All methods that highlight RS have their strengths and weaknesses then similar trend obtained from different tools should increase accuracy of the results characterizing RS occurrence and the confidence of the results. Moreover, the studies to detect the occurrence of RS are generally performed with two measurement time points while in oncology clinical trials more than two assessments is usually planned. Then we need also tools for the longitudinal analysis of the potential occurrence of RS.

The intent of this study was thus to investigate statistical methods to characterize the occurrence of RS for HRQoL in breast cancer (BC) patients.

The primary objective was to assess if Multiple Correspondence Analysis (MCA), which is a factor analysis and a model of IRT named the Linear Logistic model with Relaxed Assumptions (LLRA), had convergent results with then-test method to characterize recalibration component of RS.

The secondary objective was to assess if Principal Component Analysis (PCA), which is another factor analysis model, could be a valuable tool to longitudinally identify the reconceptualization and reprioritization components of RS independently of the occurrence of the recalibration component of RS.

## Methods

### Patients and eligibility criteria

A prospective, multicenter, randomized cohort study was performed in the cancer care centers at Dijon, Nancy, and the university hospitals of Strasbourg and Reims (cities of France). It is a collaboration between different teams with complementary skills and an interest in the topic and all these teams are involved in quality of life research. All women initially hospitalized between February 2006 and February 2008 for diagnosis or treatment of primary or suspected BC were eligible for inclusion. We anticipated that patients with no confirmed BC could constitute a control group. Nevertheless, due to the low effective (less than 10% of patients included) they could not constitute a larger control group then they were excluded from the analyses. Women with cancer other than BC, already undergoing BC treatment, or with a previous history of cancer were excluded. Written informed consent was obtained from every participant and the protocol was approved by Dijon University Hospital Ethics committee [20].

### Health-related quality of life assessment

HRQoL was evaluated using the EORTC QLQ-C30 and EORTC QLQ-BR23 BC specific tool at four time points: at baseline (initial examination or initial hospitalization), at discharge following initial hospitalization, at three months (M3) and six months (M6) [21,22]. The QLQ-C30 and its BC module BR23 are validated tools in assessing HRQoL in cancer, specifically in BC [21,22]. The QLQ-C30 comprises 30 items and measures five functional scales (physical, role, emotional, cognitive and social functioning), global health status (GHS), financial difficulties and eight symptom scales (fatigue, nausea and vomiting, pain, dyspnea, insomnia, appetite loss, constipation, diarrhea) [22]. The BR23 module comprises 23 items that generate four functional scales (body image, sexual functioning, sexual enjoyment, future perspective) and four symptom scales (systemic therapy side effects (STSE), breast symptoms, arm symptoms, upset by hair loss) [22].

Response categories vary from 1 to 4 on a Likert scale for the QLQ-C30 and BR23 questionnaires, with 1 corresponding to the best state for functional scales or no symptoms, and 4 corresponding to the worst state for functional scales or the highest symptomatic level. For sexual dimensions, the response categories are reversed. Scores are generated according to the EORTC Scoring Manual [23]. These scores vary from 0 (worst) to 100 (best) for the functional dimensions and GHS, and from 0 (best) to 100 (worst) for the symptom dimensions.

A five-point difference in EORTC HRQoL scores is considered as the minimal clinically important difference (MCID) [24].

### Then test assessment

In this study, the retrospective pre-test/post-test design was used to detect recalibration [13]. At each follow-up time point, one prospective and one retrospective measurement were performed. The retrospective assessments at the end of initial hospitalization and at M3 refer to baseline HRQoL. At M6, the retrospective measurement refers to HRQoL at M3. The order of the then-test and post-test of HRQoL questionnaires was randomized with a 1:1 allocation and stratification by center to assess the impact of the order on RS occurrence and estimate. In arm A, the order of the questionnaires was post-test/then-test. In arm B, the order was then-test/post-test. Authorization was obtained from the EORTC HRQoL unit to adapt the HRQoL questionnaires (EORTC QLQ-C30 and module BR23) to the then-test assessment. The impact of the retrospective or prospective administration of the questionnaire on RS occurrence has already been analyzed in a previous paper showing no order effect and is not treated in the present paper [20].

Treatments as well as clinical and sociodemographic variables were recorded at inclusion.

### Statistical analyses

#### Studied population and missing data

Variables collected at baseline were described with median and range for continuous variables and percentage for qualitative variables, with percentage of missing data. No imputation was performed on missing items. Scores were calculated if at least half of the items were answered according to the recommendations of the EORTC scoring manual [23]. No imputation was performed on missing scores.

MCA and LLRA were both performed on patients with all items of the studied dimension (each dimension of the QLQ-C30 and the QLQ-BR23) filled out at the then-test and the pre-test measurement time points and with a MCID between then-test and pre-test of at least 5 points for the given dimension. This selection was done in order to retain a clinically meaningful difference of the recalibration occurrence.

PCA were performed on patients with all scores available at the four prospective measurement times for one questionnaire (QLQ-C30 or BR23).

For each analysis, patients retained were compared to those excluded according to baseline characteristics in order to check the random missing data profile and then a possible selection bias.

#### Recalibration

For each score, the mean difference (MD) between each then-test and the corresponding pre-test was calculated and described as mean (SD). The existence of a significant recalibration was tested with a Wilcoxon matched pairs test. The effect size was calculated in order to assess the magnitude of RS effect and was defined as the mean change score between the then-test and the corresponding pre-test dividing by the standard deviation of patients at the prospective measurement time.

The primary objective was to assess if MCA and the LLRA model of IRT had convergent results with the then-test method to characterize the recalibration component of RS.

Firstly, recalibration was thus explored by MCA [25]. MCA is a factor analysis dedicated to qualitative variables and can identify links between categories of polytomous variables. This method is thus well adapted to the items constructed on a Likert scale. This analysis was applied to items of each dimension according to the Then-test method, i.e. with pre-test and then-test measures of the same HRQoL. Only recalibration was explored with this method since only one dimension was included. Therefore, recalibration was confirmed by a correlation between two different response categories of the same item measured at pre-test and at then-test measurement time [26]. The study was limited to the first two axes.

LLRA, a IRT model for measuring change, was then applied to explore recalibration [27-30].

IRT and Classical Test Theory differ in terms of score calculation. Classical Test Theory is mainly based on observed scores while in IRT, item responses play the key role: IRT models the item responses to the latent trait by a probabilistic model. The raw score is thus not considered as a good representation of the latent trait but the response to each item is considered directly. The relationship between the observed score and the latent trait is no longer linear. They are generally linked and modeled by a logistic function. The IRT models introduce the concepts of item easiness parameters and person parameters.

The person parameter corresponds to the level of the patient on the latent trait (e.g. the level of HRQoL). The item parameter is the location of the item on the latent trait and corresponds to a level of difficulty or easiness in this model.

The LLRA requires neither items’ unidimensionality nor distributional assumptions about the population of subjects [31]. In addition, the LLRA can fit with polytomous responses and was developed in order to measure the change occurring between several measurement time points [32]. To give up the unidimensionality of the items, items have to be measured at two measurement time points or more [32,33].

The main idea of the LLRA model is not to consider longitudinal change as a change in person parameters, but rather as a change in item parameters. In this way, person parameters are fixed over time and only item parameters vary. Since person parameters are nuisance parameters, we can estimate the item parameter trend instead of the person parameter trend by conditional maximum likelihood [34]. Indeed, fewer parameters have to be estimated and they did not depend on the sample considered.

One item I with parameter βi evaluated twice on an individual can be seen as a pair of virtual items I*1 and I*2 with two item parameters β*i1 and β*i2 respectively. For the pre-test, β*i1 = βi while for the then-test β*i2 = βi + τ where τ is the upward or downward trend effect of item easiness parameter. This parameter is targeted by LLRA [35]. In cases of polytomous items, for each item with (m + 1) response categories there are m category parameters. The trend parameter τ is the same for each category parameter. The design matrix was constructed such that there is one trend parameter for each item. If possible, the trend was generalized for all items of a dimension. The general form of LLRA, a longitudinal IRT model adapted to polytomous items, is based on the partial credit approach [35].

A positive (or negative) trend τ for one item implies that the item easiness parameter increases (or decreases) at the time of the then-test measurement compared to the pre-test measurement. Patients choose higher (or lower) response categories in the retrospective then-test measure than in the prospective one for this item. In this way, recalibration would be indicated by a significant positive or negative trend for one dimension.

Convergent results between MCA and IRT would correspond to:

a significant positive trend parameter for IRT and some upward recalibration profiles highlighted by MCA (i.e. patients choose upper response categories at the then-test assessment as compared to the prospective measurement time) more than some downward recalibration profiles (patients choose lower response categories at the then-test assessment as compared to the prospective measurement time).

a significant negative trend parameter for IRT and some downward recalibration profiles highlighted by MCA (patients choose lower response categories at the then-test assessment) rather than some upward recalibration profiles (patients choose upper response categories at the then-test assessment).

an insignificant trend parameter for IRT and well-balanced recalibration profiles highlighted by MCA (as many patients choose higher than lower response categories at the retrospective measurement time as compared to the prospective measure).

GHS was excluded from MCA and LLRA because of the high number of response categories. There are seven response categories for both items measuring GHS. To apply a longitudinal model of IRT, all seven categories have to be represented at each measurement time point, which was not the case in the present study. GHS was excluded from MCA in order to be consistent with IRT.

#### Reprioritization and reconceptualization

The secondary objective was to assess if PCA could be a valuable tool to longitudinally identify the reconceptualization and reprioritization components of RS independently of the occurrence of recalibration component of RS.

PCA was performed on patients with all scores available at all prospective measurement times and for one questionnaire (QLQ-C30 or BR23) on the scores generated for all dimensions of each prospective questionnaire [12,14,15,36]. PCA was performed only for one questionnaire in order to have clear and understandable graphs. Reprioritization was indicated by a change in scores generating the first two principal components: scales generating the first principal component are a priority to patients while those generating the second principal component are secondary. Changes occurring at the first principal component are considered as major and those occurring at the second principal component as minor. In this way, changes were qualified in the first axis of “major reprioritization” and in the second axis of “secondary reprioritization”. The study was limited to the first two principal components, according to the Scree test [37]. Reconceptualization was reflected by a change in the structure of the graph of correlations between scores and principal components, as well as in the connection or opposition of some scores. Concerning the module BR23, sexual enjoyment and hair loss were excluded from the analysis given the number of missing values.

All analyses were performed with R software [38] using FactoMineR library for factor analyses [39] and eRm library for LLRA [34,35,40].

The statistical significance level was reduced to p = 0.002 for all analyses in order to prevent false positive results due to the number of multiple comparisons performed (alpha risk 0.05 divided by the number of dimensions analyzed).

## Results

### Patients

Between February 2006 and February 2008, 381 patients were included in the four participating centers. Mean age was 58.4 (standard deviation = 11) years. Three hundred and forty (89%) patients had confirmed BC. Complete clinical and pathologic features of the population are given in Table 1.

**Table 1:** Baseline patient characteristics

	**N**	**%**
**Hospital**		
Dijon	271	71.1
Nancy	74	19.4
Reims	18	4.7
Strasbourg	18	4.7
**Inclusion criteria**		
Confirmed primary breast cancer	242	63.5
Suspicion of primary breast cancer	138	36.2
Unknown	1	0.3
**Cancer**		
Confirmed	340	89.2
Not confirmed	38	10.0
Unknown	3	0.8
**Lymph node dissection(LND)**		
Axillary LND	138	36.2
Sentinel lymph node biopsy	131	34.4
ALND + SLNB	32	8.4
No LND	75	19.7
Unknown	5	1.3
**Surgery type**		
Mastectmoy	124	32.6
No mastectomy	241	63.3
Unknown	16	4.2
**Chemotherapy**		
Yes	155	40.7
No	218	57.2
Unknown	8	2.1
**Radiotherapy**		
Yes	254	66.7
No	119	31.2
Unknown	8	2.1
**Hormone therapy**		
Yes	170	44.6
No	203	53.3
Unknown	8	2.1
**Questionnaires order**		
Arm 1: then-test/post-test	192	50.4
Arm 2: post-test/then-test	189	49.6

### HRQoL questionnaires completion and missing data

Table 2 describes the number of completed QLQ-C30 and BR23 questionnaires at each measurement time.

**Table 2:** Description of the EORTC QLQ-C30 and BR23 questionnaires received at each measurement time

	**QLQ-C30**		**QLQ-BR23**	
	**Then-test**	**Post-test**	**Then-test**	**Post-test**
Baseline		359 (94.2%)		357 (93.7%)
After surgery	347 (91.1%)	347 (91.1%)	347 (91.1%)	346 (90.8%)
3 months	339 (90.0%)	342 (89.8%)	355 (87.9%)	340 (89.2%)
6 months	314 (82.4%)	322 (84.5%)	313 (82.1%)	322 (84.5%)

317 (93%) patients had at least one HRQoL score at baseline, 311 (91%) on discharge following initial hospitalization (i.e. after surgery), 304 (89%) at M3 and 290 (85%) at M6.

Median time for HRQoL assessments between baseline and the discharge following initial hospitalization was 6 days, range [1.5; 81.5].

Patients retained for MCA and LLRA with a 5-point MCID were similar to those excluded according to baseline characteristics for each analysis (data not shown). Patients retained for PCA with all the four prospective measurement times were similar to those excluded except that they seem to be older (data not shown).

### Recalibration

After surgery (Table 3), the recalibration effect was statistically and clinically significant for emotional functioning (MD = 5.36) and future perspectives (MD = 7.41) dimensions with a moderate effect size (0.21 and 0.24 respectively). At M3, the recalibration effect was statistically and clinically significant for role (MD = -6.50), emotional (MD = 6.97) and social functioning (MD = -5.01), insomnia (MD = -6.93), body image (MD = -8.16) and future perspectives (MD = 6.95) dimensions.

**Table 3:** Recalibration component of response shift effect assessed with the then-test method at each measurement time

	**Baseline HRQoL**		**Then-test 1 minus pre-test**				**Then-test 2 minus pre-test**				**HRQoL at three months**		**Then-test 3 minus pre-test**			
	**N**	**Mean (SD)**	**N**	**Mean (SD)**	***P***	**Effect size**	**N**	**Mean (SD)**	***P***	**Effect size**	**N**	**Mean (SD)**	**N**	**Mean (SD)**	***P***	**Effect size**
**QLQ-C30**																
Global Health Status	310	68.66 (20.52)	280	-0.80 (16.67)	0.600	-0.04	275	**-4.21 (18.45)**	**<0.001**	**-0.21**	300	60.02 (20.19)	266	0.91 (21.30)	0.48	0.05
Physical functioning	313	90.01 (15.50)	283	-0.50 (10.78)	0.987	-0.03	280	-1.59 (13.26)	0.053	-0.11	301	81.31 (16.76)	273	**5.10 (14.52)**	**<0.001**	**0.31**
Role functioning	309	89.28 (20.38)	281	-1.90 (18.70)	0.182	-0.09	282	**-6.50 (23.72)**	**<0.001**	**-0.32**	300	74.06 (30.16)	269	**8.55 (28.99)**	**<0.001**	**0.30**
Emotional functioning	308	64.86 (26.20)	279	**5.36 (18.64)**	**<0.001**	**0.21**	280	**6.97 (21.48)**	**<0.001**	**0.27**	300	72.80 (22.54)	270	-2.85 (24.55)	0.054	-0.11
Cognitive functioning	313	83.23 (20.76)	280	2.80 (14.97)	0.027	0.13	281	2.37 (18.27)	0.041	0.11	301	82.84 (21.11)	239	4.03 (20.80)	0.004	0.17
Social functioning	307	90.34 (18.88)	264	-0.51 (16.18)	0.644	-0.03	276	**-5.01 (20.70)**	**<0.001**	**-0.27**	298	81.37 (25.42)	266	**6.02 (25.77)**	**<0.001**	**0.22**
Fatigue	310	22.89 (22.92)	278	-1.48 (18.22)	0.039	-0.06	279	1.75 (20.92)	0.228	0.08	300	32.82 (24.08)	270	**-11.03 (25.36)**	**<0.001**	**-0.43**
Nausea and vomiting	312	3.53 (11.18)	270	-0.77 (8.34)	0.130	-0.07	282	1.77 (15.11)	0.092	0.16	299	3.44 (10.90)	269	-3.22 (19.95)	0.010	-0.18
Pain	316	12.45 (20.87)	285	0.53 (19.04)	0.897	0.02	283	3.24 (23.03)	0.032	0.15	304	25.08 (24.82)	274	**-6.02 (23.98)**	**<0.001**	**-0.23**
Dyspnea	310	11.72 (31.87)	280	-2.02 (15.19)	0.036	-0.09	279	-1.08 (15.58)	0.333	-0.05	301	12.86 (20.89)	269	-3.59 (24.08)	0.009	-0.15
Insomnia	307	38.11 (31.87)	277	-5.30 (27.14)	0.003	-0.17	274	**-6.93 (30.94)**	**<0.001**	**-0.22**	299	36.63 (30.27)	266	**-5.64 (32.59)**	**0.002**	**-0.18**
Appetite loss	312	11.75 (22.46)	280	-3.45 520.35	0.005	-0.15	280	-1.19 (23.75)	0.323	-0.05	299	10.20 (20.13)	267	-4.62 (25.02)	0.004	-0.18
Constipation	310	12.47 (22.80)	276	-1.09 (21.15)	0.520	-0.05	277	1.56 (24.93)	0.284	0.07	298	21.38 (30.87)	264	-4.29 (26.61)	0.006	-0.16
Diarrhea	309	8.63 (16.48)	278	-2.88 (12.66)	<0.001	-0.17	277	-2.89 (17.25)	0.010	-0.17	296	4.76 (12.30)	263	-0.63 (22.07)	0.483	-0.04
Financial difficulties	300	4.56 (14.60)	264	0.38 (12.83)	0.741	0.03	269	0.99 (16.51)	0.453	0.07	299	5.86 (15.93)	264	-2.15 (18.14)	0.048	-0.10
**QLQ-BR23**																
Body image	295	90.04 (17.32)	262	-0.76 (11.95)	0.505	-0.05	257	**-8.16 (16.96)**	**<0.001**	**0.48**	302	70.76 (30.77)	269	**7.78 (24.82)**	**<0.001**	**0.25**
Sexual functioning	274	76.46 (24.01)	232	-0.50 (13.82)	0.207	-0.02	222	-1.21 (15.92)	0.206	-0.05	267	79.65 (22.06)	224	-4.69 (18.52)	0.002	-0.21
Sexual enjoyment	126	43.92 (28.79)	99	2.36 (11.91)	0.124	0.09	100	3.33 (22.97)	0.284	0.12	138	52.17 (29.31)	108	-1.54 (23.41)	0.548	-0.06
Future perspective	295	47.46 (30.86)	261	**7.41 (30.60)**	**<0.001**	**0.24**	259	**6.95 (32.47)**	**<0.001**	**0.23**	301	54.49 (32.76)	269	-0.12 (33.02)	0.968	-0.01
STSE	308	13.29 (15.30)	280	-1.71 (9.89)	0.008	-0.11	271	0.73 (13.84)	0.563	0.05	301	25.13 (20.20)	271	**-4.76 (19.52)**	**<0.001**	**-0.24**
Breast symptoms	273	11.25 (14.92)	243	-0.73 (14.39)	0.152	-0.05	239	2.15 (19.76)	0.379	0.14	302	24.73 (22.97)	273	**-7.28 (20.70)**	**<0.001**	**-0.31**
Arm symptoms	297	8.06 (14.42)	268	1.58 (18.49)	0.572	0.11	261	2.43 (16.53)	0.011	0.17	302	16.39 (18.54)	273	-2.71 (17.70)	0.010	-0.15
Hair loss	55	32.73 (36.57)	34	0.98 (17.38)	0.749	0.03	31	1.08 (25.07)	0.506	0.03	131	53.69 (39.78)	54	-8.03 (40.92)	0.173	-0.21

P: Wilcoxon matched test *P* value.

SD: standard deviation.

Results in bold correspond to clinically and statistically significant results.

At M6, the recalibration effect was statistically and clinically significant for physical (MD = 5.10), role (MD = 8.55) and social functioning (MD = 6.02) and for fatigue (MD = -11.03), pain (MD = -6.02), insomnia (MD = -5.64), body image (MD = 7.78) and breast symptoms (MD = -7.28).

### Recalibration and MCA

All results obtained on the QLQ-C30 and QLQ-BR23 are summarized in Table 4 and in Table 5, respectively. Qi_k (resp. Ri_k) refers to the k-th response category of the i-th item of a prospective (resp. retrospective) questionnaire on the graph.

**Table 4:** Main recalibration profiles highlighted by a multiple correspondence analysis performed on the EORTC QLQ-C30

**Dimension (items)**	**Time points**	**N**	**Percentage of recalibration (number of patients)**	**Recalibration category 1 to category 2**	**Recalibration category 2 to category 1**	**Recalibration category 3 to category 4**	**Recalibration category 4 to category 3**	**Categories 3 and 4 dispersed**
Physical functioning	T1 - T2_R^a^	100	37% (272)	Q1-Q5	Q1-Q5			Q1-Q5
(Q1, Q5)	T1 - T3_R^b^	139	51% (274)	Q1-Q5	Q1-Q5			Q1-Q5
	T3 - T4_R^c^	201	76% (266)	Q1-Q5	Q1-Q5			Q1-Q5
Role functioning	T1 - T2_R	84	31% (272)	Q6, Q7	Q6, Q7			Q6, Q7
(Q6-Q7)	T1 - T3_R	118	43% (274)	Q6, Q7	Q6, Q7	Q6, Q7	Q6, Q7	
	T3 - T4_R	164	63% (261)	Q6, Q7	Q6, Q7			Q6, Q7
Emotional functioning	T1 - T2_R	180	68% (263)	Q21-Q24	Q21-Q24			
(Q21-Q24)	T1 - T3_R	208	79% (263)	Q21-Q24	Q21-Q24			
	T3 - T4_R	196	77% (255)	Q21-Q24	Q21-Q24			Q21-Q24
Cognitive functioning	T1 - T2_R	103	39% (266)	Q20, Q25				
(Q20, Q25)	T1 - T3_R	129	48% (268)	Q20, Q25	Q20, Q25	Q20, Q25	Q20, Q25	Q20, Q25
	T3 - T4_R	130	51% (256)	Q20, Q25	Q20, Q25	Q20, Q25	Q20, Q25	
Social functioning	T1 - T2_R	75	29% (260)	Q26, Q27	Q26, Q27			Q26, Q27
(Q26, Q27)	T1 - T3_R	101	38% (268)	Q26, Q27	Q26, Q27	Q26, Q27	Q26, Q27	
	T3 - T4_R	142	56% (255)	Q26, Q27	Q26, Q27	Q26, Q27	Q26, Q27	Q26, Q27
Fatigue	T1 - T2_R	141	54% (259)	Q10, Q12, Q18	Q10, Q12, Q18			Q10, Q12, Q18
(Q10, Q12, Q18)	T1 - T3_R	160	61% (261)	Q10, Q12, Q18	Q10, Q12, Q18	Q12	Q10, Q12, Q18	Q10, Q12, Q18
	T3 - T4_R	183	73% (251)	Q10, Q12, Q18	Q10, Q12, Q18		Q10, Q12, Q18	Q10, Q12, Q18
Nausea and vomiting	T1 - T2_R	37	13% (280)	Q14	Q14, Q15			Q14, Q15
(Q14, Q15)	T1 - T3_R	65	24% (275)	Q14	Q14		Q14	
	T3 - T4_R	98	37% (265)	Q14	Q14			
Pain	T1 - T2_R	96	36% (267)	Q9	Q9			Q9, Q19
(Q9, Q19)	T1 - T3_R	124	46% (268)	Q9, Q19	Q9, Q19			Q9, Q19
	T3 - T4_R	148	58% (253)	Q9, Q19	Q9, Q19		Q9, Q19	
Insomnia	T1 - T2_R	115	42% (277)	Q11	Q11		Q11	
(Q8)	T1 - T3_R	135	49% (274)	Q11	Q11	Q11	Q11	
	T3 - T4_R	147	55% (266)	Q11	Q11	Q11	Q11	
Dyspnea	T1 - T2_R	50	18% (280)	Q8	Q8		Q8	
(Q11)	T1 - T3_R	55	20% (279)	Q8	Q8	Q8	Q8	
	T3 - T4_R	94	35% (269)	Q8	Q8	Q8	Q8	
Appetite loss	T1 - T2_R	61	22% (280)	Q13	Q13		Q13	
(Q13)	T1 - T3_R	85	30% (280)	Q13	Q13		Q13	
	T3 - T4_R	90	34% (267)	Q13	Q13		Q13	
Constipation	T1 - T2_R	71	26% (276)	Q16	Q16	Q16	Q16	
(Q16)	T1 - T3_R	89	32% (277)	Q16	Q16	Q16	Q16	
	T3 - T4_R	93	35% (264)	Q16	Q16	Q16	Q16	
Diarrhea	T1 - T2_R	39	14% (278)	Q17	Q17			
(Q17)	T1 - T3_R	64	23% (277)	Q17	Q17			
	T3 - T4_R	61	24% (263)	Q17	Q17		Q17	
Financial difficulties	T1 - T2_R	22	8% (264)	Q28	Q28			
(Q28)	T1 - T3_R	33	12% (269)	Q28	Q28		Q28	
	T3 - T4_R	47	18% (264)	Q28	Q28		Q28	

Only patients with a recalibration of 5-point at least between a pre-test and a then-test measure are incorporated in these analyses. Items with observed recalibration are listed.

^a^T1 → T2_R: comparison of baseline HRQoL assessment and retrospective measure performed after surgery.

^b^T1 → T3_R: comparison of baseline HRQoL assessment and retrospective measure performed three months later.

^c^T3 → T4_R: comparison of HRQoL assessment at three months and retrospective measure performed three months later.

As example, 100 patients presented a significant recalibration of physical functioning among the 272 patients with all the five items of the dimension answered at both measurement time. The graph representing the response categories highlight some recalibration profile:

- patients who had chosen response category 1 at the prospective measurement time for all the 5 items and who had chosen response category 2 to the same items at the retrospective measurement time.

- patients who had chosen response category 2 at the prospective measurement time for all the 5 items and who had chosen response category 1 to the same items at the retrospective measurement time.

- no recalibration profile is highlighted for response categories 3 and 4 and few patients had chosen these categories of response (these response categories are dispersed).

**Table 5:** Main recalibration profiles highlighted by multiple correspondence analysis performed on the QLQ-BR23 for patients with recalibration

		**N**	**Percentage of recalibration (number of patients retained)**	**Recalibration category 1 to category 2**	**Recalibration category 2 to category 1**	**Recalibration category 3 to category 2**	**Recalibration category 3 to category 4**	**Recalibration category 4 to category 3**	**Categories 3 and 4 dispersed**
Body image	T1-T2_R^a^	68	29% (236)	Q9-Q12	Q9-Q12			Q9, Q10	Q9-Q12
(Q9-Q12)	T1-T3_R^b^	119	50% (238)	Q9-Q12	Q9-Q12		Q9, Q12		Q9-Q12
	T3-T4_R^c^	153	63% (244)	Q9, Q10	Q9, Q10				Q9-Q12
Sexual functioning	T1-T2_R	55	25% (219)	Q14		Q14			
(Q14, Q15)	T1-T3_R	71	34% (210)	Q14		Q14, Q15			
	T3-T4_R	78	37% (213)	Q14, Q15	Q14, Q15	Q14, Q15			
Sexual enjoyment	T1-T2_R	13	13% (99)	Q16	Q16	Q16	Q16	Q16	
(Q16)	T1-T3_R	39	39% (100)	Q16	Q16	Q16	Q16	Q16	
	T3-T4_R	33	31% (108)	Q16	Q16	Q16	Q16	Q16	
Future perspectives	T1-T2_R	122	47% (261)	Q13	Q13	Q13	Q13	Q13	
(Q13)	T1-T3_R	135	52% (259)	Q13	Q13		Q13	Q13	
	T3-T4_R	144	54% (269)	Q13	Q13		Q13	Q13	
Systemic therapy side effects	T1-T2_R	55	26% (209)						
(Q1-Q4, Q6-Q8)	T1-T3_R	61	32% (190)						
	T3-T4_R	77	39% (200)						
Breast symptoms	T1-T2_R	114	51% (223)	Q20-Q23	Q20-Q23				Q20-Q23
(Q20-Q23)	T1-T3_R	135	61% (223)	Q20-Q23	Q20-Q23	Q20-Q23		Q20-Q23	Q20-Q23
	T3-T4_R	190	74% (258)	Q20-Q23	Q20-Q23	Q20-Q23			Q20-Q23
Arm symptoms	T1-T2_R	97	38% (255)	Q17-Q19	Q17-Q19				Q17-Q19
(Q17-Q19)	T1-T3_R	111	46% (244)	Q17-Q19	Q17-Q19			Q17	Q17-Q19
	T3-T4_R	156	60% (260)	Q17	Q17-Q19				Q17-Q19
Hair loss	T1-T2_R	6	18% (34)	Q5		Q5		Q5	
(Q5)	T1-T3_R	14	45% (31)	Q5		Q5	Q5	Q5	
	T3-T4_R	24	44% (54)	Q5	Q5		Q5	Q5	

Only patients with a recalibration of 5-point at least between a pre-test and a then-test measure are incorporated in these analyses. Items with observed recalibration are listed.

^a^T1 → T2_R: comparison of baseline HRQoL assessment and retrospective measure performed after surgery.

^b^T1 → T3_R: comparison of baseline HRQoL assessment and retrospective measure performed three months later.

^c^T3 → T4_R: comparison of HRQoL assessment at three months and retrospective measure performed three months later.

**Figure 1:**
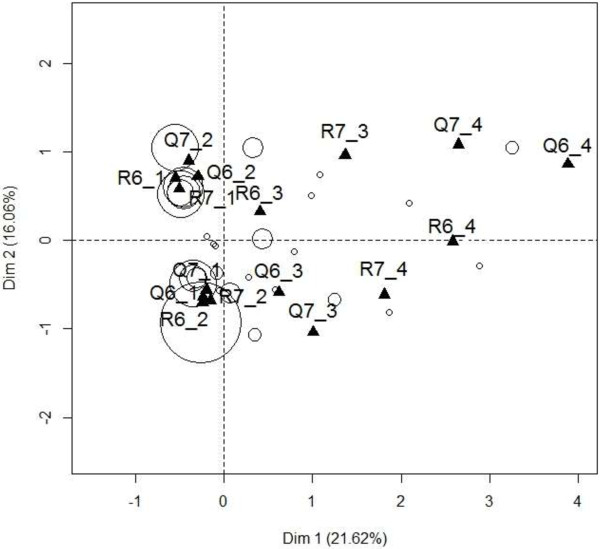
**Variable response categories according the first two axes obtained by Multiple Correspondence Analysis between prospective measure of role functioning at baseline and retrospective measure after surgery (N = 84).** Questions 6 and 7 of the EORTC QLQ-C30 measure this dimension: Qi_k (or Ri_k) refers to the k-th category of the i-th item of a prospective (or retrospective) questionnaire on the graph. Patients are represented by circles that are proportional to the number of patients with the same coordinates (i.e. who had given the same responses).

Figure 1 presents the graph obtained for baseline and the then-test performed after surgery for role functioning. 272 patients answered the items 6 (Were you limited in doing either your work or other daily activities?) and 7 (Were you limited in pursuing your hobbies or other leisure time activities?) measuring the role functioning scale at baseline and at the retrospective measurement after surgery referring to the baseline HRQoL. Response categories are coded “1/2/3/4” respectively for “Not at all/A little/Quite a bit/Very much”. Among these patients, 84 (31%) had a MCID of at least 5 points between the two measures. Figure 1 highlights two main patterns of recalibration: patients who had reported an excellent role functioning at baseline (i.e. had chosen response category 1 for both items 6 and 7 at baseline) and who had declared a slightly worse role functioning level when they reevaluated this dimension retrospectively after surgery (i.e. chose response category 2 for both items 6 and 7 at the retrospective measurement time), and vice versa (i.e. had chosen response category 2 for both items 6 and 7 at baseline and had chosen response category 2 for both items 6 and 7 at the retrospective measurement time). The first profile is suggested by an association between Q6_1, Q7_1, R6_2 and R7_2. The reverse profile corresponds to the association between Q6_2, Q7_2, R6_1 and R7_1. Recalibration profiles are less explicit for patients who had reported a low role functioning at baseline (i.e. had chosen response category 3 or 4 for both items 6 and 7 at baseline). Indeed, these patients are fewer and they did not follow a unique recalibration profile. Patients who had reported a relatively low role functioning at baseline (i.e. had chosen response category 3 for both items 6 and 7 at baseline) either tended to revise their opinion upwards or downwards by choosing either response category 4 or response category 2 for both items 6 and 7 at the retrospective assessment after surgery.

### Recalibration and IRT using LLRA

Positive trend parameters (τ = +) indicated that at the pre-test measurement, patients had overestimated their functional level or had underestimated their symptomatic or sexual level.

Based on the first retrospective reassessment of their baseline HRQoL (Table 6), patients had significantly underestimated their emotional (τ = -0.62, p < 0.001) and cognitive functioning (τ = -1.15, p < 0.001) and their level of arm symptoms (τ = 0.64, p < 0.001). Patients also had overestimated the presence of insomnia (τ = -0.49, p = 0.001) and diarrhea (τ = -1.34, p < 0.001).

**Table 6:** Trend τ of item easiness parameter estimated by linear logistic model with relaxed assumptions for each quality of life dimension

**Dimension**	**Items**	**T1 → T2_R**^**a**^			**T1 → T3_R**^**b**^			**T3 → T4_R**^**c**^		
		**N**	**τ**	**p**	**N**	**τ**	**p**	**N**	**τ**	**p**
QLQ-C30										
Physical functioning	1 – 4^d^	100	-0.02	0.858	139	0.27	0.004	201	-0.84	<0.001
Role functioning	6, 7	84	0.36^e^	0.011	118	0.71	<0.001	164	-0.60	<0.001
Emotional functioning	21 - 24	180	-0.62	<0.001	208	-0.65	<0.001	196	0.22	<0.001
Cognitive functioning	20, 25	103	-1.15	<0.001	129	-0.29	0.020	130	-0.53	<0.001
Social functioning	26, 27	75	0.07	0.791	101	0.66	<0.001	142	-0.51	<0.001
Fatigue	10, 12, 18	141	-0.19	0.280	160	0.23	0.019	183	-0.99	<0.001
Nausea and vomiting	14, 15	37	-0.60	0.022	65	0.50	0.029	98	-0.36	0.003
Pain	9, 19	96	0.11	0.410	124	0.37	0.001	148	-0.54	<0.001
Dyspnea	8	50	-0.60	0.025	55	-0.30	0.248	94	-0.42	0.014
Insomnia	11	115	-0.49	0.001	135	-0.49	<0.001	147	-0.36	0.005
Appetite loss	13	61	-0.59	0.004	85	-0.14	0.401	90	-0.51	0.003
Constipation	16	71	-0.16	0.392	89	0.17	0.295	93	-0.41	0.009
Diarrhea	17	39	-1.34	<0.001	64	-0.67	0.005	61	-0.09	0.641
Financial difficulties	28	22	0.16	0.630	33	0.25	0.322	47	-0.45	0.053
QLQ-BR23										
Body image	9 - 12	68	0.10	0.489	119	0.83	<0.001	153	-0.66	<0.001
Sexual functioning	14, 15	55	0.06	0.950	71	0.33	0.078	78	0.77	0.010
Sexual enjoyment	16	13	-1.20	0.04	39	-0.43	0.14	33	0.19	0.49
Future perspective	13	122	-0.56	<0.01	135	-0.46	<0.01	144	0.01	0.95
STSE	1 - 4; 6 - 8	55	-0.40	<0.01	61	0.21	0.013	77	-0.74	<0.001
Breast symptoms	20 - 23	114	-0.11	0.07	135	0.36	0.01	190	-0.76	<0.001
Arm symptoms	17 - 19	97	0.64	<0.001	167	0.86	<0.001	156	-0.37	0.001
Hair loss	Q5	6	0.22	0.738	16	0.12	0.808	24	-0.33	0.149

^a^T1 → T2_R: comparison of baseline quality of life assessment and retrospective measure performed after surgery.

^b^T1 → T3_R: comparison of baseline quality of life assessment and retrospective measure performed three months later.

^c^T3 → T4_R: comparison of quality of life assessment at three months and retrospective measure performed three months later.

^d^Q5 was excluded because of the patients’ responses to this item at baseline: all patients chose either category 1 or 2 for this item at baseline, whereas all four categories were represented on retrospective measures.

^e^τ =0.364: patients significantly chose higher response categories at the retrospective measure of baseline HRQoL performed after surgery.

Based on the second retrospective reassessment of their baseline HRQoL, patients had significantly overestimated their role (τ = 0.71, p < 0.001) and social functioning (τ = 0.66, p < 0.001), their body image (τ = 0.83, p < 0.001) and insomnia level (τ = -0.49, p < 0.001) and had underestimated their level of pain (τ = 0.37, p = 0.001), and arm symptoms (τ = 0.86, p < 0.001).

Regarding HRQoL at M3, patients had significantly underestimated their physical (τ = -0.84, p < 0.001), role (τ = -0.60, p < 0.001), cognitive (τ = -0.53, p < 0.001) and social functioning (τ = -0.51, p < 0.001) as well as their body image (τ = -0.66, p < 0.001). Patients also had overestimated their emotional functioning (τ = 0.22) and their levels of fatigue (τ = -0.54), pain (τ = -0.54), arm (τ = -0.37) and breast (τ = -0.76) symptoms (p ≤ 0.001).

To summarize, after surgery, the recalibration effect was statistically significant for 6/22 dimensions of the QLQ-C30 and BR23 according to the IRT model while for the then-test method it was only clinically significant for 2 of these dimensions (emotional functioning and future perspective). At M3, the recalibration effect was statistically significant for 7/22 dimensions of the QLQ-C30 and BR23 according to the IRT model and to the then-test method except for the arm symptoms (MD = 2.43, p = 0.011). At M6, the recalibration effect was statistically significant for 11/22 dimensions of the QLQ-C30 and BR23 according to the IRT model. The same results were observed for the then-test method except for the emotional (p = 0.054) and cognitive functioning (p = 0.004) and the arm symptoms (p = 0.010). A significant and clinically recalibration was also observed according to the classical then-test method for insomnia (MD = -5.64, p = 0.002) and not according to the IRT model (p = 005).

### Reprioritization and reconceptualization with PCA

PCA was performed on each prospective measure performed at baseline, post-surgery, and at M3 and M6, on patients with all scores available at the prospective measurement time for one questionnaire (QLQ-C30 or QLQ-BR23).

For the QLQ-C30 (respectively QLQ-BR23), 192 (50.4%) patients (respectively 154 (40.4%)) were retained with all scores available at the four prospective measurement times.

**Figure 2:**
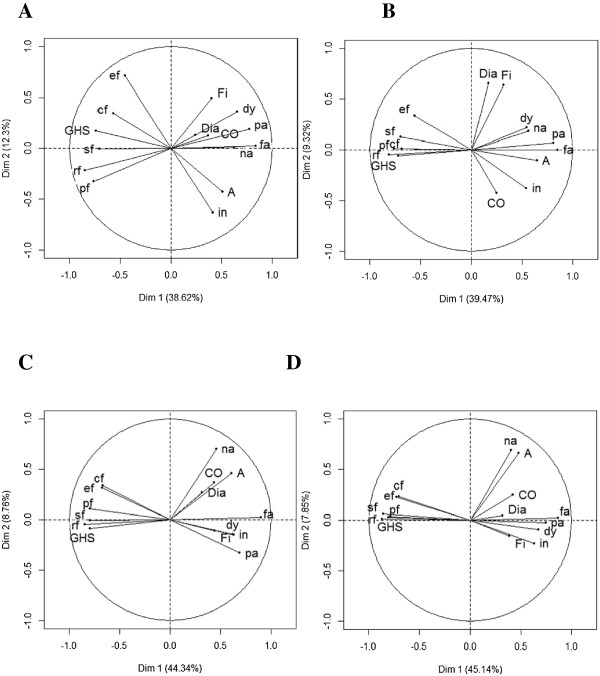
**Graph representing the correlation between QLQ-C30 scores and the first two principal components of Principal Component Analysis at each prospective measurement time (N = 192): at baseline (Panel A), just after surgery (Panel B), at three months (Panel C) and at six months (Panel D).** The QLQ-C30 measures five functional scales (physical functioning (pf), role functioning (rf), emotional functioning (ef), cognitive functioning (cf), social functioning (sf)), global health status (GHS), financial difficulties (Fi) and eight symptom scales (fatigue (fa), nausea and vomiting (na), pain (pa), dyspnea (dy), insomnia (in), appetite loss (A), constipation (CO), diarrhea (Dia)).

Concerning the QLQ-C30 (Figure 2), functional scales became more interrelated and related to the first principal component, reflecting a strong positive correlation between these scales (Table 7). This is observed at each measurement time point. Fatigue and pain remained strongly correlated at each measurement time point, a little less at M3. Diarrhea and financial difficulties were correlated just after surgery (Figure 2B). Nausea and vomiting were correlated to appetite loss at M6 (Figure 2D).

**Table 7:** Correlation between quality of life scores and the first two first axis of principal component analysis on prospective measures

**Scores**	**T1: baseline**		**T2: after surgery**		**T3: 3 months**		**T4: six months**	
	**First axis**	**Second axis**	**First axis**	**Second axis**	**First axis**	**Second axis**	**First axis**	**Second axis**
QLQ-C30 (N = 192)								
GHS	-0.74	0.17	-0.72	-0.06	-0.79	-0.09	-0.79	0.03
Physical functioning	-0.76	-0.33	-0.76	0.01	-0.79	0.11	-0.81	0.03
Role functioning	-0.85	-0.22	-0.81	-0.05	-0.85	-0.05	-0.87	0.01
Emotional functioning	-0.46	0.71	-0.56	0.33	-0.68	0.31	0.73	0.23
Cognitive functioning	-0.57	0.34	-0.69	0.01	-0.67	0.34	-0.70	0.23
Social functioning	-0.71	-0.01	-0.70	0.13	-0.79	-0.01	-0.86	0.06
Fatigue	0.84	0.03	0.85	-0.01	0.90	0.02	0.87	0.02
Nausea and vomiting	0.63	0.01	0.56	0.18	0.46	0.70	0.40	0.69
Pain	0.77	0.19	0.81	0.07	0.69	-0.33	0.75	-0.02
Dyspnea	0.66	0.35	0.55	0.22	0.62	-0.14	0.67	-0.09
Insomnia	0.41	-0.63	0.54	-0.38	0.63	-0.15	0.63	-0.23
Appetite loss	0.51	-0.43	0.65	-0.11	0.61	0.46	0.48	0.66
Constipation	0.36	0.12	0.25	-0.42	0.44	0.37	0.42	0.25
Diarrhea	0.24	0.13	0.17	0.66	0.32	0.27	0.32	0.04
Financial difficulties	0.40	0.49	0.31	0.64	0.44	-0.11	0.38	-0.16
QLQ-BR23 (N = 154)								
Body image	-0.70	0.47	-0.64	0.45	-0.79	0.07	-0.79	0.25
Sexual functioning	0.30	-0.44	0.24	-0.52	0.31	-0.51	0.41	-0.41
Future perspective	-0.31	0.46	-0.53	0.52	-0.76	0.25	-0.73	0.30
Systemic therapy side effects	0.78	-0.01	0.72	0.28	0.61	-0.49	0.67	-0.21
Breast symptoms	0.58	0.49	0.64	0.38	0.56	0.66	0.57	0.64
Arm symptoms	0.66	0.49	0.75	0.32	0.69	0.44	0.63	0.57

Reprioritization was mainly secondary, as it mostly affected the second principal component: fatigue and pain were still priority symptoms at each prospective measure, since they were still highly correlated with the first principal component at each prospective measure. These symptoms mainly affected physical, social and role functioning as well as GHS. At M6, all functional scales were affected by these symptoms. The second axis highlighted secondary symptoms, namely insomnia at baseline, diarrhea after surgery, nausea and vomiting at M3 and M6 (Table 7).

**Figure 3:**
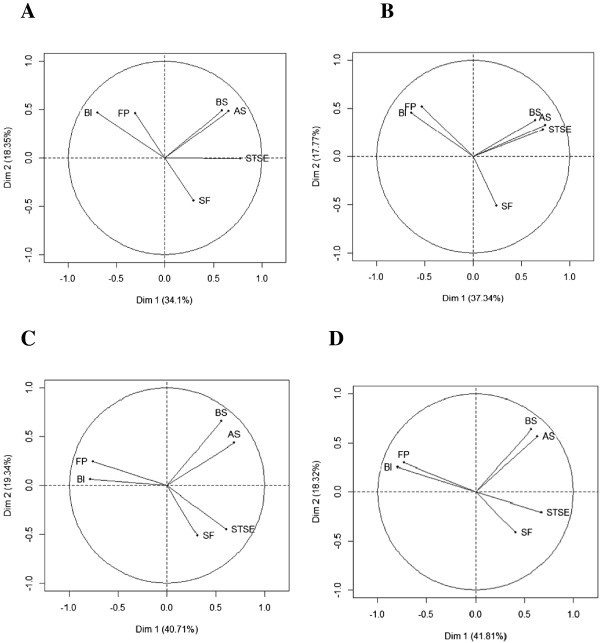
**Graph representing the correlation between QLQ-BR23 scores and the first two principal components of Principal Component Analysis at each prospective measurement time (N = 154): at baseline (Panel A), just after surgery (Panel B), at three months (Panel C) and at six months (Panel D).** The QLQ-BR23 measures four functional scales (body image (BI), sexual functioning (SEXF), sexual enjoyment (SE), future perspective (FP)) and four symptom scales (systemic therapy side effects (STSE), breast symptoms (BS), arm symptoms (AS), upset by hair loss (HL)). SE and HL are excluded from these analyses.

Concerning the QLQ-BR23, STSE affected the patients’ body image since these dimensions remained strongly negatively correlated (Figure 3). These scales were highly correlated with the first principal component (Table 7). Post-surgery, arm symptoms as well as body image and STSE were significantly correlated with the first principal component. Thus, arm symptoms were equally important to body image and STSE regarding patient HRQoL level. At M3 and M6, future perspectives became significantly correlated with the first principal component and thus gained importance, highlighting a major reprioritization. Breast symptoms and sexual functioning were not significantly associated with the two first axes at baseline: they were minor dimensions. After surgery, only sexual functioning was a relevant factor, while at M3 and M6, only breast symptoms were relevant.

Reconceptualization is illustrated by changes in correlations (positive or negative) between variables at each measurement time point. At each measurement time point, body image score was opposed to STSE score. Post-surgery, STSE was associated with arm symptoms. At M3 and M6, a high body image score was associated with high future perspective.

## Discussion

The present study demonstrates that response shift effect occurred in patients with primary breast cancer, just after surgery, as well as at 3 and 6 months. The intent of this study was to investigate statistical methods to characterize the occurrence of response shift in breast cancer patients.

Our primary objective was to assess if MCA and IRT model had convergent results with the then-test method to characterize recalibration component of RS.

Both methods explored are convergent to the then-test method. When the then-test method highlighted a clinically significant recalibration, MCA highlighted a general trend to overestimate or underestimate their HRQoL level choosing higher or lower response categories according to the direction of the recalibration effect. IRT model showed a statistically significant general trend (positive or negative) of item easiness parameter with the exception of insomnia at 6 months for which a recalibration is not detected by IRT at the alpha level p = 0.002 but borderline (p = 0.005). When the mean difference between the then-test and the pre-test is not significant, i.e. no clinically significant recalibration occurs, MCA highlighted as many patients recalibrate upward than downward their HRQoL level and then there is not a general trend to overestimate or underestimate their HRQoL level. The IRT model also highlighted that the trend of item easiness parameter is not significant. However, some discrepancies are observed: for 4/6 dimensions for which a recalibration was detected by IRT and not significant according to the then-test method at the first retrospective assessment, 1/7 at the second one, and 3/11 at the last retrospective assessment. Thus, the IRT model detects more recalibration effect than the classical then-test method.

MCA and IRT models highlight convergent results. Based on the retrospective assessment of baseline HRQoL after surgery and according to the LLRA, the trend of item easiness parameter is insignificant for social functioning. The corresponding MCA shows readjustment between response categories 1 and 2 and between response categories 3 and 4. In this way, as many patients had chosen higher than lower response categories at the retrospective measurement time as compared to the baseline measure, which is consistent with the LLRA. Based on retrospective assessment of baseline HRQoL at 3 months and according to the LLRA, patients had overestimated their body image with a positive trend of item easiness parameter. Regarding the corresponding MCA, it highlights a readjustment from response categories 2 to response categories 3 and from response categories 3 to response categories 4. In this way, patients had chosen higher response categories at the retrospective measurement time compared to the prospective measure indicating an overestimation of body image at baseline. Based on the retrospective assessment of the three months HRQoL at 6 months, patients had overestimated their pain level with a negative trend of item easiness parameter according to the LLRA. Regarding the MCA performed on pain at the same measurement times, it shows a recalibration between response categories 1 and 2, and only from response categories 4 to 3, not from 3 to 4.

The secondary objective was to assess if PCA could be a valuable tool to longitudinally identify the reconceptualization and reprioritization components of RS independently of the occurrence of recalibration component of RS. PCA indicated a reprioritization of the HRQoL domains as evaluated by the QLQ-C30. Patients’ anxiety probably related to the diagnosis of cancer and surgery seemed to be a major concern at baseline before the start of treatment, along with insomnia, which generated the second principal component, after fatigue and pain, which generated the first principal component. After surgery, diarrhea symptoms increased in importance, reflecting the impact of treatment. These results underline how patients adapt to their disease. At 3 months and 6 months, nausea and vomiting were more important as compared to diarrhea, also reflecting the toxicities of cancer treatment, especially chemotherapy. Regarding the QLQ-BR23, patients with a high level of systemic therapy side effects after surgery also tended to report a high level of arm symptoms, which can be due to the recent surgery. From 3 months, arm symptoms become less important, while future perspectives gained importance for primary breast cancer patients. Our results indicate that there is no correlation between breast symptoms and sexual functioning at 3 and 6 months.

No reprioritization was observed for the QLQ-C30 and QLQ-BR23 between the measures at M3 and M6. Patients seemed to assess their HRQoL with the same relative importance at 6 months as they did at 3 months suggesting that after treatment initiation they have a more “stabilized” appreciation of HRQoL dimensions.

The reprioritization of symptomatic scales enables interpretation of HRQoL levels and changes and impact of treatments and disease on HRQoL. Then based on these results we suggest that the occurrence of the reprioritization component of RS should be taken into account in the interpretation of the results of the longitudinal analysis. Deterioration of a scale, which becomes more important over time for the patient, could have a strong impact on patient’s overall HRQoL level and could indicate priority for care. Conversely, deterioration of a scale, which for the patient loses importance over time, could have a minor impact on patient’s general HRQoL level.

Reconceptualization is reflected by changes in connections and contrasts between variables, and more generally by changes in graph structure of PCA. The functional scales of the QLQ-C30 became increasingly interrelated. When one functional scale is affected by cancer treatment or disease progression, then it is likely that all the other functional scales are affected. Moreover, patients had associated nausea and vomiting to appetite loss at 6 months.

These results suggest that PCA is an indirect method in investigating the reprioritization and reconceptualization components of RS.

The main limitation of this work is the use of the Then-test as the standard method to explore recalibration. The Then-test method is increasingly called into question [41-43], mainly because it can induce a recall bias [13]. Indeed, the second reassessment of baseline HRQoL was three months after baseline and the reassessment of HRQoL at M3 was three months after the prospective measure so it may induce a recall bias.

Schwartz et al. have proposed some guidelines to improve the stringency of the Then-test method [41]. In their paper, Schwartz et al. recommended to include a control group, which would not susceptible to RS. As RS is a treatment-dependent phenomenon, we tried to constitute a control group including patients with only a suspicion of BC. However, the number of patients with no confirmed BC was not sufficient to constitute a control group. Others explanations of detection of RS effect could then also be formulated as social desirability responding. This hypothesis cannot be verified since a control group could not be constituted. As it was recommended by Schwartz et al., we reported effect sizes for recalibration in order to assess the magnitude of RS effect. A Bonferroni correction of type I error rate was performed in order to minimize false positive conclusions. The guidelines also recommended to use internal or external validation approaches as performance-based, perception-based, and evaluation-based items/subscales for internal validation of then-test results and clinical measures indicating health state at baseline and follow-up for external validation. However, we failed to include such approaches in this present study. The instructions of the retrospective questionnaires clearly indicate the patients to think back to the referent time as advisable by Schwartz et al. Moreover, the nomenclature used in this paper to characterize the recalibration component of RS is those recommended [41]. The Then-test method is based on the assumption that patients rate their HRQoL post-test and pre-test levels with the same criteria, since the assessments occur at the same time point. A test of the measurement invariance of the Then-test method would be necessary in this study in order to validate the then-test and to assess the possible recall bias due to its retrospective nature. This would be planned in another analysis using the Oort’s procedure [16,44].

Based on this study, substituting the then-test with the LLRA and MCA to explore the recalibration component of RS cannot be recommended at this time. Nevertheless, IRT using LLRA could reinforce the Then-test method because of the improved interpretation of recalibration. This model is effective, and the results are clearer, more explicit and easy to summarize and to interpret. These methods should be used in other studies to validate their ability to reinforce the then-test method.

SEM is often used nowadays to demonstrate RS [11,16,17,45-48]. These models are not dependent on the Then-test method. However, they are based on the raw score and not on the items. In this way, IRT as compared to SEM could be more informative. Moreover, at this time, SEM has never been applied to the EORTC HRQoL questionnaires in order to highlight occurrence of the response shift effect.

It would be interesting to compare the statistical method described in the present study (factor analysis and IRT) to SEM applied on prospective measure in another paper in order to check their ability to capture all the three components of RS. There is a need to investigate all these methods using simulated data in order to establish differences using these three methods.

Factor analysis presents the advantage of graphically exploring all the components of RS. This visual representation is interesting in order to explore reconceptualization, which is the most conceptual component of the RS effect. Moreover, at this time, few methods have been proposed to identify this component [16] and in our point of view no gold standard has emerged. In addition, no additional questionnaires are required for exploring reconceptualization and reprioritization. Thus, the use of PCA on the scores of the main questionnaires seems to be adequate in exploring these components. SEM is also often used to assess these components. However, our objective was to investigate the PCA method already used in the past [14,15,36] and not to apply SEM. Indeed, PCA is a special case of SEM.

IRT models and factor analysis are mostly used in the development and validation of HRQoL questionnaires [49-55]. However, several studies have begun to use IRT in longitudinal studies of HRQoL [29,56-61], underscoring the potential of these models in longitudinal analyses. Moreover, longitudinal IRT model was used in order to characterize recalibration component of RS. Few studies have investigated RS using IRT while differential item functioning based on IRT was also proposed as alternative approach [18,62,63].

Finally, PCA were performed on patients with all scores available at all the prospective measurement times. Only 40% to 50% of patients were thus retained in the analysis but these patients were comparable to those excluded according to baseline characteristics except there was an age effect which may reflect a selection bias.

The data presented in this article confirm the potential of IRT models in longitudinal HRQoL studies, especially their ability to characterize more precisely the recalibration component of RS. Our data also underline the interest of PCA to characterize reprioritization and reconceptualization components of RS. These results confirm the need to take recalibration into account when comparing longitudinal HRQoL data between patient groups and the need to explore the other components in order to better interpret results [64,65]. The items of these questionnaire are prone to response shift effect since they are evaluation-based items. Then an objective assessment by the patient cannot be made. Despite the fact that items of these questionnaires are prone to RS effect. Some work is still needed to provide both a longitudinal analysis method easy to understand for the clinician and to extract the potential measurement bias due to the occurrence of a response shift effect. Another solution would be to develop or use other questionnaires not prone to response shift effect with more performance-based items [41]. Future studies should investigate the ability of these statistical methods to capture all components of RS without the then-test method.

## Abbreviations

BC: Breast cancer; GHS: Global Health Status; EORTC: European Organization for Research and Treatment of Cancer; HRQoL: Health-related quality of life; IRT: Item response theory; LLRA: Linear logistic model with relaxed assumptions; MCA: Multiple Correspondence Analyses; MCID: Minimal clinically important difference; MD: mean difference; PCA: Principal component analyses; RS: Response shift; SEM: Structural equation modeling; STSE: Systemic therapy side effects.

## Competing interests

The authors declare that they have no competing interests.

## Authors’ contributions

AA performed the statistical analyses and interpretation and written the manuscript, CBM interpreted the data and drafted the manuscript, TC, FG, MV, DJ, MM, SC, JC, OG designed the study, SC, JC, OG included the patients, ZH interpreted the data, FB designed the study, written protocol, managed the statistical analyses, interpreted the data and review the draft. All authors read and approved the final manuscript.
